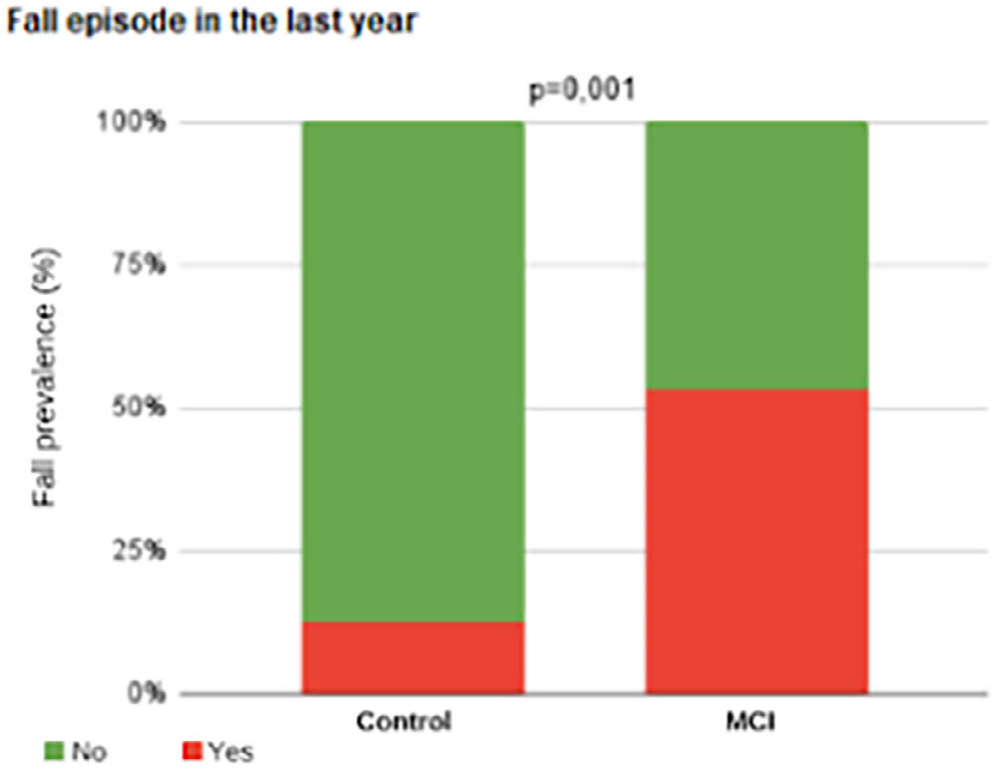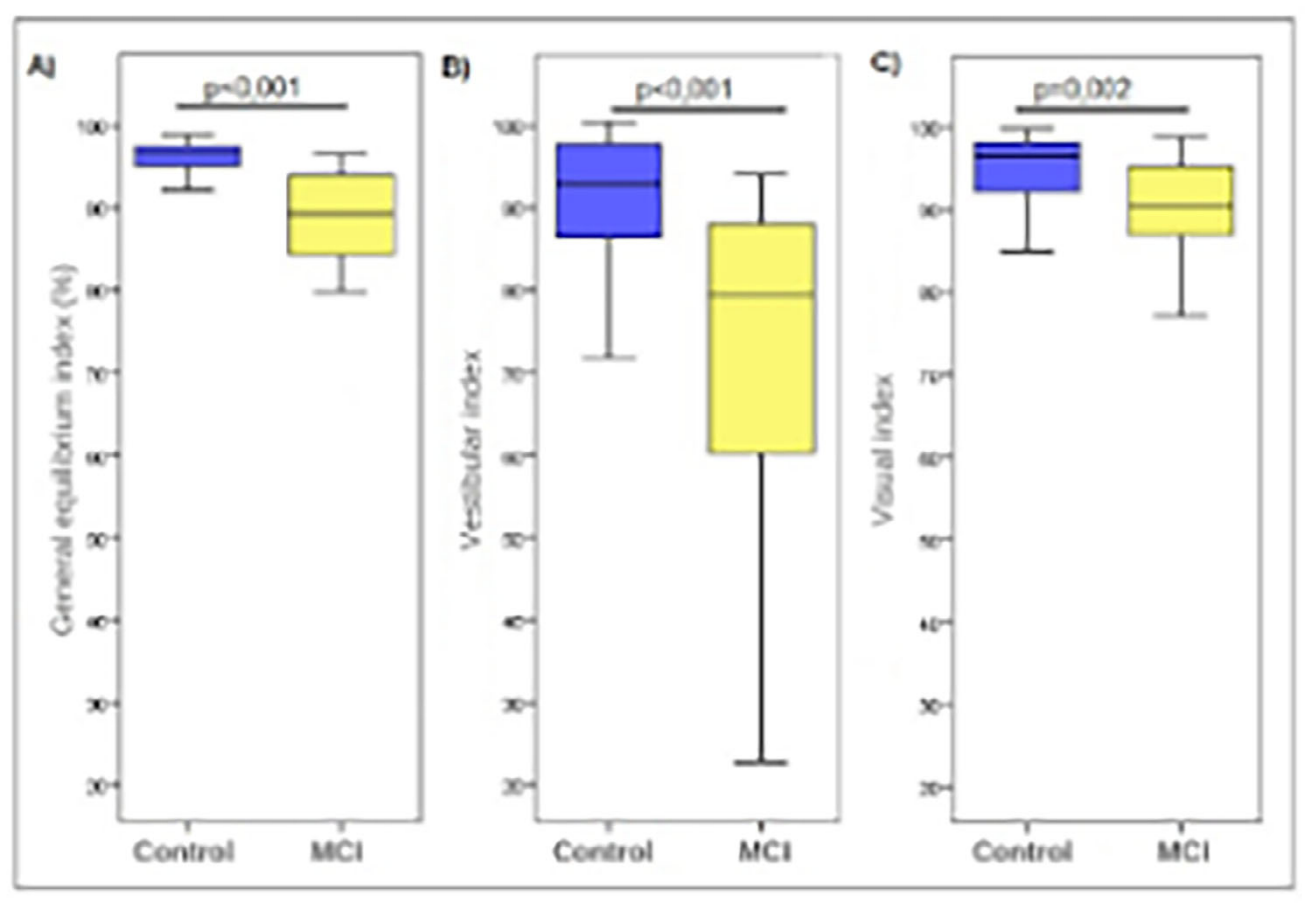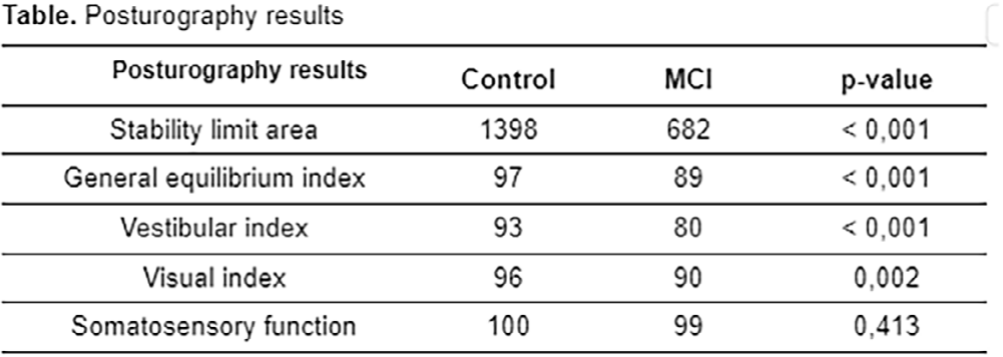# Posturographic findings and reports of falls in elderly people with mild cognitive impairment: a comparative study

**DOI:** 10.1002/alz.093060

**Published:** 2025-01-09

**Authors:** Michele da Rocha Anselmo, Juliana de Sá Novaes, Gabriela Tomé Oliveira Engelmann, Bernardo M Viana, Maria Aparecida Camargos Bicalho, Ludimila Labanca, Ana Julia Delfim de Oliveira

**Affiliations:** ^1^ Universidade Federal de Minas Gerais, Belo Horizonte, Minas Gerais Brazil; ^2^ Cog‐Aging Research Group, Universidade Federal de Minas Gerais (UFMG), Belo Horizonte, Minas Gerais Brazil; ^3^ Universidade Federal de Minas Gerais, Belo Horizonte Brazil; ^4^ Molecular Medicine Postgraduate Program, School of Medicine, Universidade Federal de Minas Gerais (UFMG), Belo Horizonte, Minas Gerais Brazil; ^5^ Older Adult’s Psychiatry and Psychology Extension Program (PROEPSI), School of Medicine, Universidade Federal de Minas Gerais (UFMG), Belo Horizonte, Minas Gerais Brazil; ^6^ Jenny de Andrade Faria Institute – Outpatient Reference Center for the Elderly, Universidade Federal de Minas Gerais (UFMG), Belo Horizonte, Minas Gerais Brazil; ^7^ Federal University of Minas Gerais, Belo Horizonte, Minas Gerais Brazil; ^8^ Geriatrics and Gerontology Center Clinical Hospital of University of Minas Gerais, Belo Horizonte, Minas Gerais Brazil; ^9^ Cog‐Aging Research Group, Belo Horizonte, Minas Gerais Brazil; ^10^ Department of Clinical Medicine, Faculdade de Medicina, Universidade Federal de Minas Gerais, Belo Horizonte, Minas Gerais Brazil; ^11^ National Institute of Science and Technology Neurotec R (INCT‐MM), Faculdade de Medicina, Universidade Federal de Minas Gerais, Belo Horizonte Brazil; ^12^ Department of Psychiatry, School of Medicine, Federal University of Minas Gerais, Belo Horizonte, Minas Gerais Brazil; ^13^ Older Adult’s Psychiatry and Psychology Extension Program I Federal University of Minas Gerais, Belo Horizonte, Minas Gerais Brazil; ^14^ UFMG, Belo Horizonte Brazil

## Abstract

**Background:**

Changes in the connections between the cortical regions responsible for integrating balance are observed in individuals with MCI, however, studies that clarify the association of these changes and the risk of falling due to body imbalance are still rare. The present study aims to compare the posturography response of individuals diagnosed with MCI in relation to individuals in a control group without MCI.

**Methods:**

This research project was approved by the local ethics committee. The sample was composed of 64 elderly people, of which 32 participants were from the MCI group and 32 from the control group. Participants underwent medical and neuropsychological evaluation where the characterization of general health and cognitive diagnosis designation was determined. Furthermore, an anamnesis composed of questions regarding the presence of complaints of dizziness, reports of fall episodes in the last year and the presence of other associated comorbidities was applied. The Timed‐Up and Go scale was used and static posturography was performed to determine the postural profile of the sample. To evaluate posturography, two tests were performed: stability limit test and sensory integration test. The data from the present study were tabulated in Microsoft Excel® version 10.0 and analyzed using SPPS® version 20 software.

**Results:**

Participants were comparable in terms of age, body weight, gender and education. The CCL group showed increased TUG time compared to the control group (p = 0.001) indicating decreased mobility. The occurrence of 53% of reports of falls in the last year was observed in the CCL group, with this presentation being higher than that observed in the control group (p = 0.001). In the posturography results, impairment was found in the CCL group in relation to the control at the limit of stability (p≤0.001), general balance index (p≤0.001), visual index (0.002) and vestibular index (<0.001).

**Conclusions:**

Elderly people diagnosed with MCI may be more susceptible to balance disorders, increasing their risk of falling compared to healthy elderly people.